# Isolation, identification and screening of hidrolytic enzymes producing phylloplane yeasts

**DOI:** 10.1186/1753-6561-8-S4-P261

**Published:** 2014-10-01

**Authors:** Gilberto de Aguiar Pereira, Guilherme Teixeira Gomes, Amanda Machado Rozolem, Gisele Maria de Andrade de Nóbrega, Fernando Gomes Barcellos, Elisete Pains Rodrigues

**Affiliations:** 1 State University of Londrina, Laboratory of Genetics of Fungi, 86057-970, Londrina, Paraná, Brazil

## Background

Microorganisms are common colonizers of superficial (phylloplane) and internal tissues (endophyte) of a variety plant species [1]. Phylloplane yeasts are considered a promising source of new and interesting biotechnological activities, particularly hydrolytic enzymes; however, the diversity of yeasts colonizing plant phylloplane and is still poorly known for most plant species. Considering the immense diversity of Brazilian flora, which include many species with medicinal properties, this knowledge is even more limited. Given the above, this study objective to isolate, identify and to evaluate the production of hidrolytic enzymes of yeasts associated with leaves, stems and flowers of alecrim-do-campo (Baccharis dracunculifolia DC), a plant recognized as the main source of exudates used by bees to produce green propolis and also, for its medicinal properties.

## Methods

For isolation of yeasts, samples leaves, stems and flowers of B. dracunculifolia were colected; 10 g were macered in 90 mL of saline solution (0,85%) and dilutions of the suspension were spread in solid medium BDA and TSA. After purification and preservation, the isolates were characterized by colony morphology and grouped. Isolates representant of each group were subsequently identified by molecular techniques based on the amplification and sequence analysis of ITS1-5, 8S-ITS2 rDNA. For evaluation and screening of potential enzymes producers, enzymatic activity was performed in solid media [2,3]. Proteolitic and celulolitic activities were tested in YEPG media with 2% casein 0.5% carboxymethylcellulose (CMC). Activitities of the enzymes pectinase, amylase, lipase and esterase were performed in YNB media containing 1% pectin, 0.2% starch, 1% oil and 1% Tween 80, respectively. After growth for 3-5 days at 28 ° C the enzymatic activities of the isolates were evaluated by the production of degradation halo around the colony, which is the visual indication of hydrolysis of their respective substrates. Assays were performed with three replicates and expressed by enzymatic indice (IE), which was obtained by ration between diameter of halo and diamater of colony (mm).

## Results and conclusions

Were obtained 69 isolates, being 74% of these isolates of yeasts and yeast-like fungi. On the basis of on colony morphology the isolates were grouped into four morphotypes (white, brown, black or salmon colony). The majority of the isolates was originating from samples of flowers and showed black colonies on PDA media. By analysis of ITS1-5,8S-ITS2 rDNA sequences in Genbank database, the isolates were identified as members of the species Starmerella bombicola, Aureobasidium leucospermi, A. pullulans, Rhodotorulla mucilaginosa and Occultifur externus. The isolates were promissing for enzyme production, specially for amylolytic and lipolytic activities which were predominant among the isolates. The most promising isolates were belonging to the genus Aureobasidium sp., which showed activities for the five enzymes tested. The isolates identified as promising for the production of enzymes, especially those of the genus Aureobasidium sp. were selected for more specific analyzes aimed at quantification and perfecting of culture conditions that allow greater production and can contribute to to the development of alternatives to enzymes already available in market.
